# The Hepatoselective Glucokinase Activator PF-04991532 Ameliorates Hyperglycemia without Causing Hepatic Steatosis in Diabetic Rats

**DOI:** 10.1371/journal.pone.0097139

**Published:** 2014-05-23

**Authors:** Derek M. Erion, Amanda Lapworth, Paul A. Amor, Guoyun Bai, Nicholas B. Vera, Ronald W. Clark, Qingyun Yan, Yimin Zhu, Trenton T. Ross, Julie Purkal, Matthew Gorgoglione, Guodong Zhang, Vinicius Bonato, Levenia Baker, Nicole Barucci, Theresa D’Aquila, Alan Robertson, Robert J. Aiello, Jiangli Yan, Jeff Trimmer, Timothy P. Rolph, Jeffrey A. Pfefferkorn

**Affiliations:** 1 Cardiovascular, Metabolic & Endocrine Disease Research Unit, Pfizer Worldwide Research & Development, Cambridge, Massachusetts, United States of America; 2 Groton Center of Chemistry, Pfizer Worldwide Research & Development, Groton, Connecticut, United States of America; CRCHUM-Montreal Diabetes Research Center, Canada

## Abstract

Hyperglycemia resulting from type 2 diabetes mellitus (T2DM) is the main cause of diabetic complications such as retinopathy and neuropathy. A reduction in hyperglycemia has been shown to prevent these associated complications supporting the importance of glucose control. Glucokinase converts glucose to glucose-6-phosphate and determines glucose flux into the β-cells and hepatocytes. Since activation of glucokinase in β-cells is associated with increased risk of hypoglycemia, we hypothesized that selectively activating hepatic glucokinase would reduce fasting and postprandial glucose with minimal risk of hypoglycemia. Previous studies have shown that hepatic glucokinase overexpression is able to restore glucose homeostasis in diabetic models; however, these overexpression experiments have also revealed that excessive increases in hepatic glucokinase activity may also cause hepatosteatosis. Herein we sought to evaluate whether liver specific pharmacological activation of hepatic glucokinase is an effective strategy to reduce hyperglycemia without causing adverse hepatic lipids changes. To test this hypothesis, we evaluated a hepatoselective glucokinase activator, PF-04991532, in Goto-Kakizaki rats. In these studies, PF-04991532 reduced plasma glucose concentrations independent of changes in insulin concentrations in a dose-dependent manner both acutely and after 28 days of sub-chronic treatment. During a hyperglycemic clamp in Goto-Kakizaki rats, the glucose infusion rate was increased approximately 5-fold with PF-04991532. This increase in glucose infusion can be partially attributed to the 60% reduction in endogenous glucose production. While PF-04991532 induced dose-dependent increases in plasma triglyceride concentrations it had no effect on hepatic triglyceride concentrations in Goto-Kakizaki rats. Interestingly, PF-04991532 decreased intracellular AMP concentrations and increased hepatic futile cycling. These data suggest that hepatoselective glucokinase activation may offer glycemic control without inducing hepatic steatosis supporting the evaluation of tissue specific activators in clinical trials.

## Introduction

Type 2 diabetes mellitus (T2DM) leads to an elevation in hepatic glucose production driven by insulin resistance in addition to progressive loss of insulin secretion and inappropriate elevations in glucagon concentrations [1]–[3]. There remains a continued need for novel anti-diabetic agents to help effectively control hyperglycemia and reduce micro- and macrovascular complications. Agents that increase hepatic glucose uptake and decrease hepatic glucose production could prove to be effective in controlling hyperglycemia in diabetics.

Glucokinase (also known as hexokinase IV) converts glucose to glucose-6-phosphate in the gut, hypothalamus, pancreas, and liver determining the flux of glucose into these cells [4]. Glucokinase found in the β-cells serves as a “glucostat” by controlling glucose stimulated insulin secretion (GSIS) [5]. The control strength of glucokinase on insulin secretion is illustrated by the phenotypes of human genetic variants. Gain of function mutations which activate glucokinase are associated with conditions of low blood glucose such as hyperinsulinemic hypoglycemia of infancy [6], [7] whereas loss of function variants are associated with hyperglycemia and various forms of diabetes [8], [9]. Pharmacological agents that systemically activate glucokinase have been shown to lower glucose concentrations in T2DM and healthy subjects. However, in many cases these agents have also been associated with adverse hypoglycemic risk due to increased insulin secretion at inappropriately low glucose concentrations [10], [11]. Additionally, questions have arisen regarding the durability of systemic glucokinase activation due to loss of efficacy observed in several Phase 2 clinical trials [10], [12] of systemic glucokinase activators. To date, there is no clear explanation for the tachyphylaxis of these systemic agents, but potential causes may include decreased β-cell function, increased insulin resistance and/or loss of pancreatic glucokinase expression.

To overcome the hypoglycemia risk and potentially mitigate the durability problems of systemic glucokinase activations, efforts have been undertaken to investigate liver specific activation of glucokinase as a possible therapeutic strategy [13]–[15]. Hepatic glucokinase regulates hepatic glucose uptake, and in turn, is able to reduce hepatic glucose production and increase glycogen synthesis [16]–[20]. In the liver, but not other tissues, the activity of glucokinase is regulated through an interaction with glucokinase regulatory protein (GKRP) [21]. During periods of low glucose, GKRP binds the inactive conformation of glucokinase and sequesters the enzyme to the nucleus. As intracellular glucose concentrations increase, glucokinase is released from GKRP and diffuses into the cytoplasm where it can be converted to its active form through binding to glucose. In type 2 diabetes, hepatic glucokinase activity was found to be decreased with increasing hyperglycemia in obese patients with type 2 diabetes [22]. Moreover, multiple diabetic animal models also experience loss of hepatic glucokinase activity with increasing hyperglycemia, and normalization of hepatic glucokinase activity via over expression reduces plasma glucose while also normalizing glycogen levels and restoring regulation of HGP [23]. However, while over-expression (∼6 fold) of hepatic glucokinase using an adeno-associated virus (AAV) resulted in decreased circulating glucose concentrations in rats, this over-expression also caused a significant increase in plasma triglycerides [24]. Additionally, given the liver specific role of GKRP, genetic variants in this gene can be viewed as specific modulators of hepatic glucokinase activity [21]. Several variants in GKRP are associated with both reduced fasting plasma glucose concentrations and increased plasma triglycerides [25], [26] along with reduced risk of T2DM [27].

Given the therapeutic potential of activating hepatic glucokinase as well as the potential risk of inducing adverse circulating or hepatic lipid changes, several groups have investigated the effects of small molecule activators on glucose and lipid homeostasis. To date, these studies have been largely conducted with systemic activators which increase both liver and pancreatic glucokinase activity. For example, MK-0941 which increased hepatic glucose uptake in isolated hepatocytes and increased insulin secretion in rat islets, reduced plasma glucose concentrations in healthy dogs and in an STZ/HFF mouse model. Liver and plasma triglycerides were unchanged compared to vehicle in the latter mouse model [28]. Another structurally similar glucokinase activator preserved β-cell mass and in turn prevented overt diabetes without effecting fasting plasma or hepatic triglycerides in a ZDF rat model [29]. By contrast, a recent study encompassing multiple GK activators in rodents demonstrated altered lipid metabolism that included hepatic steatosis [30]. Many of the compounds used within the latter study were not fully characterized regarding liver versus systemic activation, and at least one, GKA50, had confirmed off-target pharmacology (retinoic acid receptor (RAR) antagonist) which may have confounded the experimental results [31]. Overall these previous studies have offered conflicting data regarding the potential for small molecule glucokinase activation to perturb lipid homeostasis. The observed differences are potentially due to a variety of factors, including: (a) differences in preclinical models; (b) differences in treatment duration and target engagement; and (c) potential confounding effects of activation of glucokinase in both the liver and pancreas. To address this latter issue, herein we sought to specifically study whether liver specific pharmacological activation of hepatic glucokinase is an effective strategy to reduce hyperglycemia without causing adverse hepatic lipids changes.

As a tool for these studies, we utilized hepatoselective glucokinase activator PF-04991532, a Phase 2 clinical candidate, which is a potent glucokinase activator [EC_50_ of 80 nM in humans and 100 nM in rats] with no known off-target pharmacology. PF-04991532 has low passive permeability to minimize peripheral distribution and it is a substrate for human OATP1B1, OATP1B3 and OATP2B1, and rodent Oatp1a1 and Oatp1b2 liver transporters leading to enhanced hepatic liver drug concentrations. [32]. In Phase 1 and 2 studies in T2DM patients, PF-04991532 revealed statistically significant reduction in weighted mean daily glucose and HbA1c with a placebo-like hypoglycemia profile [33], [34]. During Phase 2 studies, HbA1c lowering efficacy was associated with an increase in plasma triglycerides with no change in cholesterol; however, hepatic triglyceride concentrations were not measured in this study. In contrast to some of the systemic activators, the efficacy with PF-04991532 appeared to be sustained out to 12 weeks; nevertheless, longer clinical studies will need to be conducted to assess the durability of hepatic glucokinase activators. Given the change observed in plasma triglycerides with PF-04991532 in the clinic, we conducted a series of preclinical studies to better understand the mechanism underlying these changes as well as the potential for changes in hepatic lipids.

## Results

**Figure 1:**
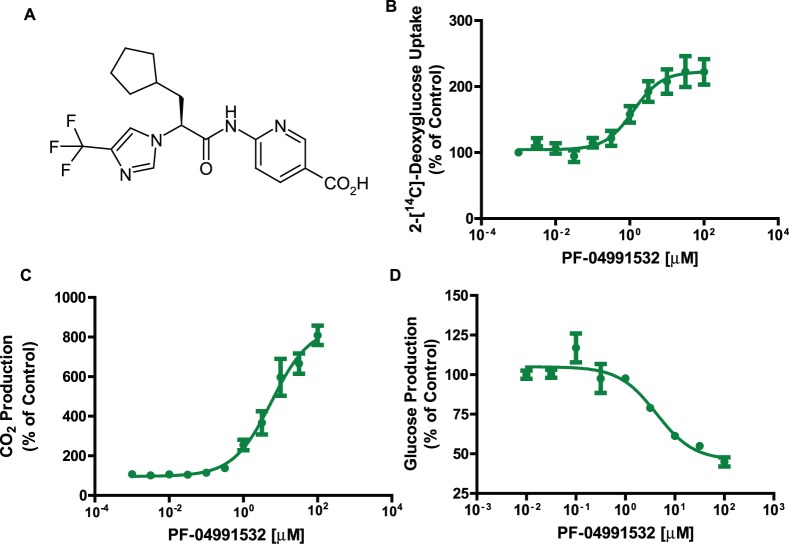
PF-04991532 regulates glucose metabolism in primary rat hepatocytes. Structure of PF-04991532 [(S)-6-(3-Cyclopentyl-2-(4-(trifluoromethyl)-1H-imidazol-1yl)propanamido)nicotinic acid] (**A**) increased glucose uptake (n = 5) (**B**), decreased glucose production from radiolabeled lactate (n = 4) (**C**), and increased CO_2_ production from glucose (n = 6) (**D**) in primary rat hepatocytes.

Mechanistic experiments conducted in freshly isolated primary rat hepatocytes treated for 1 hour with PF-04991532 (Figure 1A) showed increased 2-[^14^C]-deoxyglucose uptake (EC_50_ = 1.261 µM; Figure 1B) and increased glucose oxidation, via measuring the production of captured CO_2_ from [^14^C]-glucose (EC_50_ = 5.769 µM; Figure 1C). Additionally, PF-04991532 decreased the production of glucose from 1-[^14^C]-lactate in a dose dependent manner (EC_50_ = 0.626 µM; Figure 1D).

**Figure 2:**
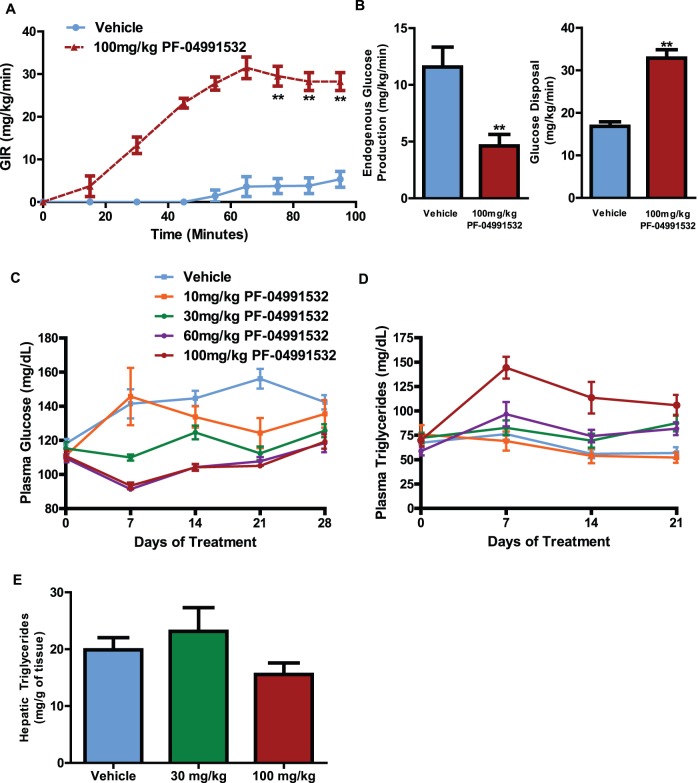
PF-04991532 improves glucose metabolism in rats. PF-04991532 increased the rate of glucose infusion in order to maintain hyperglycemia in Goto-Kakizaki rats (n = 6/group) (**A**) which can be attributed to the increased glucose disposal and decreased glucose production (n = 6/group) during steady state (**B**). PF-04991532 decreased plasma glucose in Goto-Kakizaki rats over 28 days of dosing (n = 6–8/group) [P<0.05 for 30, 60, and 100 mg/kg compared to vehicle] (**C**) which was accompanied by an increase in plasma triglycerides at the highest dose (n = 6–8/group) [P<0.05 for 100 mg/kg] (**D**). These plasma changes were not associated with any changes in liver triglycerides compared to vehicle treated animals (**E**). **P<0.01 One-way ANOVA and Tukey’s Multiple Comparison Test were used for A,C,D, & E. Student’s t-test was used for B.

Previously, sub-chronic dosing of PF-04991532 resulted in a dose dependent decrease in plasma glucose concentrations independent of any changes in insulin concentrations [32]. To compare the chronic and acute effects of PF-04991532, we subjected Goto-Kakizaki rats to an acute oral glucose tolerance test (OGTT) in which PF-04991532 showed equivalent reductions in glucose concentrations as compared to chronically treated rats for the duration of the OGTT (Figure S1A and S1B). To assess the effects of acute hepatic glucokinase activation on whole body glucose metabolism, we performed hyperglycemic clamps in Goto-Kakizaki rats. A single dose of PF-04991532 increased the glucose infusion rate in order to maintain hyperglycemia (Figure 2A). This can be attributed to a 94% increase in rate of glucose disposal and a 60% reduction in endogenous glucose production (Figure 2B). During the flux measurements, plasma glucose and insulin concentrations were unchanged during steady state (Figure S1C and S1D) consistent with the hepatoselectivity of PF-04991532 activation. During a 28 day treatment in Goto-Kakizaki rats, fasting plasma glucose concentrations were significantly reduced at the 30, 60 and 100 mg/kg doses (Figure 2C), and fasting plasma triglycerides were increased at the 100 mg/kg dose (Figure 2D). To determine the relative contribution of individual lipid species to the increase in plasma triglycerides, we analyzed the plasma using a lipidomic panel assessed by LC-MS. After a single dose, the greatest increase in plasma triglycerides was species containing 16∶1 free fatty acids. However both in the acute and chronic study, there was a general increase in all triglyceride species including triglycerides containing 18∶2 free fatty acid species (Figure S2A). Despite the elevations in plasma triglycerides, surprisingly, hepatic triglycerides in rats dosed with 19 days of PF-04991532 were identical to vehicle treated GK rats (Figure 2E). In an additional cohort treated for 28 days, we observed identical hepatic lipid concentrations between vehicle and rats dosed with PF-04991532 (Vehicle: 9.89±0.31; PF-04991532 100 mg/kg: 9.91±0.31).

**Figure 3:**
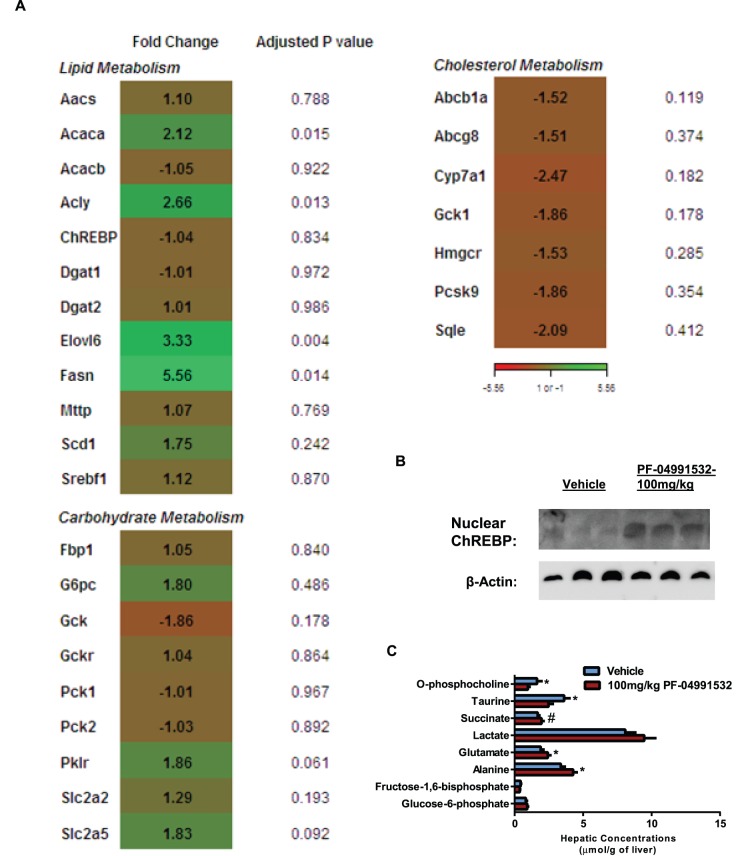
PF-04991532 effects on lipid metabolism and downstream hepatic metabolites. Expression of key lipid, carbohydrate, and cholesterol metabolism genes in rats treated with an acute 100/kg dose of PF-04991532 relative to vehicle (**A**). The increase in expression of the lipogenic genes can be explained by the increased nuclear ChREBP (n = 3/4/group) (**B**). Lastly, a metabolic profile of key metabolites using ^31^P-NMR and ^1^H-NMR in rats treated with an acute dose of PF-04991532 (n = 5–6/group) (**C**). *P<0.05; #P<0.1 for Vehicle vs. 100 mg/kg PF-04991532. Student’s t-test was used.

**Figure 4:**
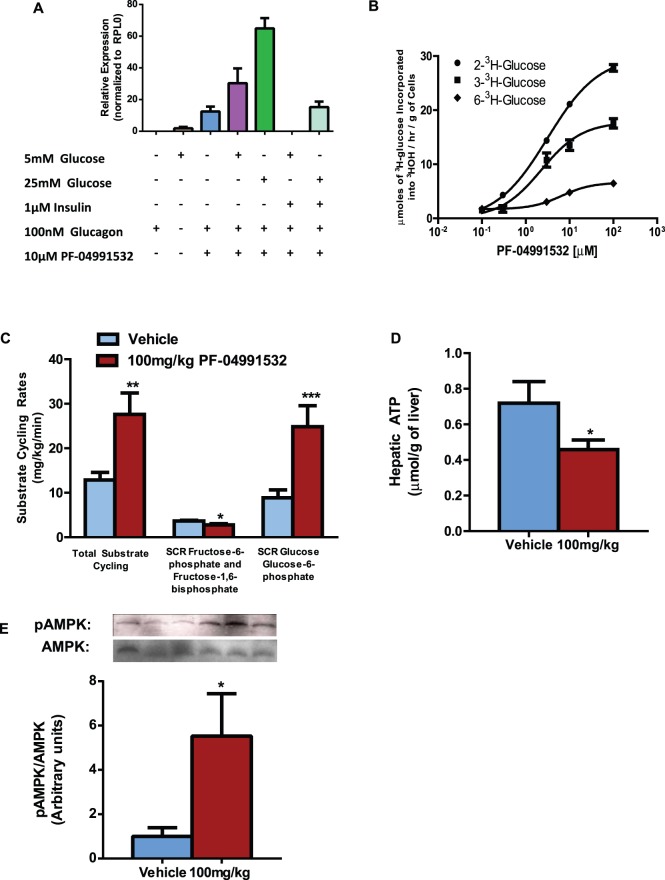
PF-04991532 increased hepatic futile cycling. The effect of hepatic glucokinase activation on G6Pase (**A**) and the loss of positional labeling (**B**) in primary rat hepatocytes. PF-04991532 increased total hepatic substrate cycling due to increased substrate cycling between glucose and glucose-6-phosphate (n = 5–7/group) (**C**). The increased substrate cycling decreased hepatic ATP concentrations as assessed by NMR (n = 6/group) (**D**) which in turn increased hepatic pAMPK/AMPK ratio (**E**) (n = 5/group). *P<0.05, **P<0.01, ***P<0.005 for Vehicle vs. 100 mg/kg PF-04991532. Student’s t-test was used.

To determine if the increases in plasma lipids could be explained by gene expression changes, we employed gene microarrays in liver samples from rats treated with a 100 mg/kg single dose of hepatic glucokinase activators. In rats treated with PF-04991532, there was increased expression of lipogenic gene expression such as acetyl-CoA carboxylase (ACC), ATP citrate lyase (ACLY), and fatty acid synthase (FAS) (Figure 3A). This was not due to differences in the expression of SREBP or ChREBP, but due to increased nuclear localization of ChREBP (Figure 3B). Additionally, we observed similar increases in FAS and ACLY gene expression after 19 days of sub-chronic treatment with PF-04991532 (Figure S2B). Lastly, PF-04991532 decreased key cholesterol biosynthetic genes such as *3-hydroxy-3-methyl-glutaryl-CoA reductase* (HMG-CoA reductase), *proprotein convertase subtilisin/kexin type 9* (PCSK9), and *squalene epoxidase*. This in turn decreased hepatic free cholesterol 12% (Figure S3A) and hepatic cholesteryl ester 14% (Figure S3B). These same rats also had increases in hepatic glutamate and alanine concentrations and surprisingly only a modest increase in hepatic glucose-6-phosphate (Figure 3C). Additionally, the microarray indicated an upregulation of hepatic *G6Pas*e, *pyruvate kinase* and down regulation of *glucokinase* indicative of increased futile cycling (Figure 3A). In isolated rat hepatocytes, PF-04991532 increased the expression of G6Pase compared to cells treated only with 100 nM glucagon, and the greatest increase in G6Pase mRNA expression was in the presence of 25 mM glucose, 100 nM glucagon and PF-04991532 (Figure 4A). In the primary rat hepatocytes treated with either 2-^3^H-glucose, 3-^3^H-glucose, or 6-^3^H-glucose, there was a dose dependent increase in the incorporation 2-^3^H-glucose and 3-^3^H-glucose into water (Figure 4B). To test the rate of substrate cycling *in vivo*, Goto-Kakizaki rats were administered a single 100 mg/kg dose of PF-04991532 and infused with 6,6-^2^H-glucose, 2-^2^H-glucose, and 3-^2^H-glucose simultaneously. The total substrate cycling rate (SCR) was increased 114% compared to vehicle treated rats than can be attributed to a 180% increase in the SCR between glucose and glucose-6-phosphate (Figure 4C). Surprisingly, hepatic glucokinase activation decreased the SCR between fructose-6-phosphate and fructose-1,6-bisphosphate (Figure 4C). The increase in futile cycling was associated with a 36% decrease in the hepatic ATP concentrations measured by NMR (Figure 4D) and a decrease in hepatic AMP;ATP ratio as measured by LC-MS (Figure S2C) which in turn led to a 5-fold increase in the phospho-AMPK to AMPK ratio (Figure 4E).

## Discussion

We found that pharmacological activation of hepatic glucokinase led to sustained reductions in plasma glucose concentrations in diabetic rats during acute and chronic dosing due to increased hepatic glucose uptake. The reduction in plasma glucose concentrations is consistent with other studies that have used an adenovirus to overexpress glucokinase [23], [24]. Although hepatic glucokinase activators increased *G6Pase* expression *in vivo* and in primary hepatocytes thus presumably increasing gluconeogenic flux, the net hepatic glucose production was decreased since the hepatic uptake of glucose was much greater than any increase in flux from glucose-6-phosphate to glucose. We observed significant glucose reductions for PF-04991532 at 100 mg/kg over 28 days, however there is a trend on day 28 for a reduction in the amount of achieved efficacy on fasting glucose concentrations with PF-04991532.

The present study also found higher hepatic concentrations of alanine and glutamate which could indicate increased amino acid synthesis that mirrors previous liver perfusion studies with glucokinase activator Piragliatin [35]. Additionally, activation of hepatic glucokinase was associated with increased plasma triglyceride concentrations with no corresponding increases in hepatic lipid content in the rodent models in these studies. Furthermore, the observed increase in plasma triglycerides is consistent with variants of GKRP, correlation of GK expression to lipogenesis in human liver samples [36] and adenovirus-mediated over expression of glucokinase in rodents [23], [24]. While taken together these studies support the notion that hepatic glucokinase regulates hepatic lipid production, our data suggest that the lipid elevations are dependent on the degree of GK activation. Given that diabetic human and diabetic rodent models have lower glucokinase activity [22], [23], restoring the activity to normal levels may be a feasible strategy that would exert significant effects on glucose concentrations while minimizing risk of elevated hepatic lipids. Additionally, the human data implicating a role for glucokinase activity in increasing lipids may be confounded by as yet uncharacterized biological roles of GKRP that function independently of glucokinase. The data presented in this study support that there is a therapeutic index to effectively control glucose concentrations without having detrimental effects on liver fat. Since triglycerides composed of 18∶2 fatty acids (dietary derived fatty acids) were elevated in rats with glucokinase activation in addition to *de novo* derived fatty acids, we hypothesize the increased plasma triglycerides in the Goto-Kakizaki are due, in part, to increased *de novo* lipogenesis that can be attributed to increased expression of key lipogenic genes and increased capacity for re-esterification of fatty acids.

The lack of change in hepatic triglyceride concentrations with PF-04991532 may have several potential explanations. First, increased hepatic AMP:ATP ratio which in turn activates AMP-activated protein kinase (AMPK) has been shown to prevent hepatic steatosis and hepatic lipid accumulation [37]. Decreased hepatic ATP concentration in rats treated with PF-04991532 may be attributed to the increased hepatic futile cycling through glucose phosphorylation and dephosphorylation. However, increased synthesis of purine molecules could also contribute to the changes in energy charge. These data are corroborated by recent results from Shiota and coworkers where ZDF rats that were treated for 14 weeks with, (S)-6-(3-cyclohexyl-2-(4(trifluoromethyl)-1H-imidazol-1-yl)propanamido)nicotinic acid, a structurally related analog of PF-04991532, in which there was no difference in hepatic triglycerides, but significant increase in plasma triglycerides [38].

Other glucokinase activators have produced conflicting results as to whether activation of glucokinase can produce efficacious glucose control without impacting hepatic lipid burden [30], [39], [40]. Since PF-04991532 is a selective liver activator, we were able to evaluate the effect of exclusively hepatic glucokinase activation. The selective activation of liver glucokinase may display unique pharmacology relative to systemic activation since liver activation can have a larger impact on hepatic futile cycling. An increase in insulin concentrations derived from activation of glucokinase in the β-cell may blunt the conversion of glucose-6-phosphate back to glucose by suppressing hepatic *G6Pase* expression and activity. Additionally, the rise in plasma insulin concentrations resulting from activation of glucokinase in the pancreas may further enhance hepatic *de novo* lipogenesis through activation of the sterol regulatory element binding protein (SREBP-1c) [41], [42].

In conclusion, PF-04991532 ameliorated hyperglycemia in a Goto-Kakizaki rat diabetic rat model by increased uptake of glucose and decreased production of glucose. Although PF-04991532 was associated with elevation in plasma triglyceride there were no changes in hepatic triglyceride in the specific rat models used in this study. These changes in plasma glucose and plasma triglyceride concentrations observed in these rodent studies are consistent with those observed in a Phase II 12-week study in type 2 diabetics using PF-04991532 [33].

## Methods

### Primary Rat Hepatocytes

Male Wistar Han rats were fasted overnight and primary hepatocytes were isolated by a two-step, in situ liver perfusion method as previously described [43]. Viability was assessed by Trypan blue and ATP levels measured with CellTiter-Glo Luminescent Cell Viability Assay (Promega, #G7570). 2-Deoxyglucose uptake, glucose oxidation, gluconeogeneis, and glucose recycling was measured in primary rat hepatocytes in suspension, incubated in Krebs Buffer containing 5.5 mM glucose, 3 mM lactate, and 300 µM pyruvate with and without PF-04991532 (100 µM- 1 nM). Primary rat hepatocytes incubated with dimethyl sulfoxide (DMSO) were used as controls. To assess glucose uptake, cells were incubated with 1 µCi [^14^C] 2-deoxyglucose (Perkin Elmer, #NEC-495A) for 15 minutes on a rotating platform. The reaction was stopped with ice cold PBS, cells washed three times, and then lysed. To measure glucose oxidation, primary rat hepatocytes were incubated with 2.5 µCi of U-[^14^C] glucose for 1 hr. Reactions were terminated by acidifying the media with 1.5 N sulfuric acid. Label incorporation into [^14^C] CO2 was determined by employing a phenethylamine trap [44]. Gluconeogenesis was measured by incubation with 0.5 µCi [^14^C]-lactate. Glucose recycling was determined by incubation with 2.5 µCi 2, 3, or 6 [^3^H] glucose. Glucose production and recycling reactions were terminated by addition of 1.5 N sulfuric acid and then neutralized with potassium hydroxide. To determine [^3^H] water production in glucose recycling experiments, samples were deproteinized with 0.4 mL of 0.3 N zinc sulfate +0.4 mL of 0.3 N barium hydroxide, pH 6–7. Separation of [^14^C] glucose and [^3^H] water was done using ion exchange columns [45].

For determining the expression of G6Pase in the primary rat hepatocytes, 50,000 freshly isolated rat hepatocytes isolated from Wistar Han rats were incubated in Williams E media overnight supplemented with 100 nM dexamethasone, 1× ITS, and 1× PenStrep. The following morning the media was aspirated, and changed to DMEM no glucose media supplemented with either 5 mM glucose, 25 mM glucose, 1 µM insulin, 100 nM glucagon, or 10 µM PF-04991532. Following 2 hours the media was aspirated, washed twice, and 100 µL of RLT was added to the cells. RNA was extracted with a Qiagen RNeasy kit (Qiagen). The abundance of transcripts was assessed by real time PCR on a 7900 Fast Real-Time PCR System (Applied Biosystems). Using TaqMan probes each run was evaluated in duplicate for both the G6Pase and Rplp0. Primers/probes were obtained from Life Technologies.

#### Hyperglycemic clamps

All procedures performed on any animals were in accordance with regulations and established guidelines and were reviewed and approved by a Pfizer Institutional Animal Care and Use Committee and all experiments within this manuscript were undertaken to minimize animal suffering during the experiment. 13 week old male Goto Kakizaki rats with in-dwelling carotid artery and jugular vein catheters were purchased from Taconic labs. Surgeries were performed one day before shipping. Upon arrival, animals were individually housed, allowed ad libitum chow, and acclimated to their new environment for 6–7 days. On the day of the experiment, PF-04991532 was weighed and resuspended in 0.5% Methyl cellulose and sonicated to make a fine suspension. A 0.5% Methyl cellulose vehicle was used in vehicle-treated rats. Animals were randomly assigned either a 100 mg/kg PF-04991532 treatment or vehicle control treatment and orally gavaged at 5 ml/kg. Studies were performed in unstressed, awake, chronically catheterized rats using the insulin clamp technique [46], [47], in combination with [3-^3^H] glucose as described previously [48]. Food was removed five hours before the infusions. All studies included a basal equilibration period lasting 90 minutes where only [3-^3^H] glucose was infused, and a post treatment period of 80 minutes. At the beginning of the basal period, infusion and sampling lines were attached to indwelling jugular and carotid catheters of rat. A continuous infusion of [3-^3^H] glucose (NEN Life Science Products; 15 µCi bolus, 0.6 µCi/kg/min) was initiated and maintained throughout the remaining 3-hours of study.

Briefly, a variable infusion of 25% glucose solution was started at time 0 and periodically adjusted to clamp the plasma glucose concentration at approximately 200 mg/dL. Plasma samples for determination of [3-^3^H] glucose were obtained at 10-minute intervals during the basal and clamp steady state periods. At 90 minutes post tracer infusion, a 5 ml/kg oral gavage of 100 mg/kg PF-04991532 or vehicle was performed. Steady state conditions for plasma glucose concentration and specific activity were achieved after 60 minutes in basal and clamp condition. At the end of the in vivo studies, rats were euthanized.

During the clamp, plasma glucose was measured by the glucose oxidase method (Analox Instruments USA, Lunenburg, MA). Plasma insulin concentrations were measured by a Mercodia ultra sensitive rat enzyme immunoassay (Alpco Diagnostics, Windham, NH). Plasma [3-^3^H] glucose radioactivity was measured in the supernatants of Ba (OH)_2_ and ZnS0_4_ precipitates of plasma samples (50 µl) after evaporation to dryness to eliminate tritiated water. Glucose concentrations of Somogyi precipitates were determined in duplicate using the glucose hexokinase method (Roche Diagnostics, Indianapolis, IN). Under steady state conditions for plasma glucose concentrations, the rate of glucose disappearance (Rd) equals the rate of glucose appearance (Ra). The latter was calculated as the ratio of the rate of infusion of [3-^3^H] glucose (dpm/min) and the steady-state plasma [3-^3^H] glucose-specific activity (dpm/mg). When exogenous glucose was given, the rate of endogenous glucose production was calculated as the difference between Ra and the exogenous glucose infusion rate (GIR, mg/kg⋅min).

#### Hepatic triglyceride measurements

Total lipids were extracted from the livers using the Folch method [49] Frozen liver aliquots (150–250 mg) were added to 4 mL ice-cold methanol (MeOH) and homogenized for 20 seconds using a Polytron homogenizer (Kinematica, Switzerland). Cold chloroform (CHCl3, 4 mL) was added and the samples homogenized for a second time for 30 seconds. The samples were transferred to extraction tubes, a further 4 mL of CHCl3 was added, and the homogenate mixed thoroughly. Samples were held at −20°C until analysis, at which point, 2.8 mL 1 M aqueous potassium chloride was added (8∶4:3 ratio CHCl3:MeOH:H2O). The samples were vortexed for 30 seconds and centrifuged at 4°C to separate the phases. The entire bottom phase was removed and dried down under nitrogen followed by resolubilization in CHCl3. All extraction solvents contained 50 µM butylated hydroxytoluene as antioxidant. Neutral lipids were isolated from the total lipid extracts using aminopropyl solid phase extraction columns as previously described [50]. The neutral lipid fraction was in turn dried down under nitrogen and resuspended in isooctane:isopropanol (98∶2). Free and esterified cholesterol, TAG and diacylglycerol were separated and detected using normal phase cyanopropyl high-performance liquid chromatography (HPLC) and a charged aerosol detector. Data are expressed as milligrams of lipid per gram (mg/g) wet weight of liver.

#### Western blotting

Livers were homogenized in 300 µL ice-cold homogenation buffer [10 mM Hepes, pH 7.4, 250 mM sucrose, 1 mM EDTA, protease inhibitor and phosphatase inhibitor (Roche Diagnostics)] with a Dounce homogenizer. The homogenate was centrifuged at 650×g for 10 min (4°C). The supernatant was transferred into the new tubes and centrifuged at 100,000×g for 20 min (4°C). The pellet (mitochondria plus nucleus) was resuspended with homogenization buffer and preserved. Protein assay was done for every fraction at the same time. 15 µg of total protein was run on SDS-PAGE, transferred to nitrocellulose membranes, and probed for ChREBP (Abcam), phospho-AMPK (Cell Signaling) and AMPK (Cell Signaling).

#### Futile cycling

Diabetic Goto-Kakizaki rats had jugular vein and carotid artery catheters placed one week before the experiment. These rats were fasted overnight, and the following morning we infused 0.45 mg/kg/min of a solution containing equal amounts of [6,6-^2^H]glucose, [2-^2^H]glucose, and [3-^2^H]glucose solution, and 0.45 mg/kg/min of unlabeled glucose for 2 hours. 100 mg/kg of PF-04991532 was administered at the start of the infusion study. All samples were measured on a Agilent 6890 GC coupled to a 5973 mass selective detector. The plasma preparation and calculations are identical to Shulman et. al. [51].

#### Gene expression analysis

Gene expression profiling of 11 liver samples (6 vehicle and 5 treated with a single 100 mg/kg dose of PF-04991532 and euthanized 3 hours post-compound dose after an OGTT) from GK rats was performed using the Rat Genome 230 2.0 Array Affymetrix chip. Quality control and pre-processing (RMA) of the hybridization signals were carried out in R-BioConductor software using the *affy* package [52]. Differential gene expression and pair-wise fold change comparisons were assessed using linear models on the log2 transformed expression values. The linear model t-statistics were regularized using the ‘moderated t’ approach of Smyth [53]. The Benjamini and Hochberg adjustment procedure [54] based on controlling the False Discovery Rate (FDR) was used to adjust for multiple independent hypotheses.

## Supporting Information

Figure S1 **Plasma glucose (A) concentrations during an OGTT immediately following the administration of PF-04991532 and after 19 days of chronic administration (B).** Plasma glucose (**C**) and insulin (**D**) concentrations during the hyperglycemic clamp.
(EPS)

Figure S2 **Plasma triglyceride species analyses by LC-MS from Goto-Kakizaki rats treated acutely with PF-04991532 or sub-chronically for 19 days with PF-04991532 (A).** Expression of FAS and ACLY after 19 days of sub-chronic treatment with PF-04991532 (**B**). Hepatic AMP:ATP ratio as assessed by LC-MS/MS. #P = 0.06 *P<0.05 vs. Vehicle. Student’s t-test was used.
(EPS)

Figure S3 **Hepatic free cholesterol (A) and cholesteryl ester (B) from Goto-Kakizaki rats treated with GK activators for 19 days.** **P<0.01 One-way ANOVA and Tukey’s Multiple Comparison Test against vehicle treated animals.
(EPS)

Methods S1 (DOCX)
