# Supplementary material for: The Hepatoselective Glucokinase Activator PF-04991532 Ameliorates Hyperglycemia without Causing Hepatic Steatosis in Diabetic Rats
Source: PLoS One. 2014 May 23;9(5):e97139. doi: 10.1371/journal.pone.0097139 (PMC4032240; doi:10.1371/journal.pone.0097139)
Supplement: Methods S1 — (DOCX) [file pone.0097139.s004.docx]

**Supplemental Methods**

*LC-MS Analyses of Plasma Triglycerides.* All rat liver tissue samples were homogenized in Methanol:Water (1:1, v/v) using the Qiagen tissue lyser. Following homogenization, lipids were extracted from the homogenate with Dichloromethane:Isopropanol:Methanol (25:10:65, v/v/v) containing the following internal standards at a concentration of 200 nM: Glyceryl Triheptadecanoate, 1,2-Dinonadecanoin, Cholesteryl Heptadecanoate, 1,2-Dilauroyl-sn-glycero-3-phosphocholine, 1-Heptadecanoyl-2-hydroxy-sn-glycero-3-phosphocholine, and Palmitoyl-L-carnitine-(N-methyl-d_3_) hydrochloride. Lipid extracts were then analyzed by UPLC-MS/MS using a Waters Acquity UPLC coupled to an AB Sciex QTRAP 5500 mass spectrometer. Lipid classes were separated by reversed-phase chromatography on a Waters Acquity UPLC BEH300 C4 column, 1.7 um, 2.1 x 50 mm. Lipid species were then analyzed on the mass spectrometer using positive ion electrospray ionization in the multiple reaction monitoring (MRM) mode. LC chromatogram peak integration was performed with AB Sciex MultiQuant software. All data reduction was performed with in-house software

*OGTT in Goto-Kakzaki Rats.* Compound was administered 60 minutes prior to the administration of a 2 g/kg glucose bolus (T = –60 min) after an overnight fast in Goto-Kakzaki rats. Blood was extracted at 0, 15, 30, 60, and 120 minutes post glucose administration by tail bleed and glucose values were measured using an AlphaTrack glucose meter.

*Nucleotides Analysis in Rat Liver Tissue*. This procedure was based on a recent publication [[1](#_ENREF_1)]. Sample Preparation. The livers were minced and homogenized in a volume of solution (methanol:10mM EDTA at pH 8.0 (80:20; v/v)) (in ml) equal to five times the weight (in g) of the tissue. The homogenate was further diluted 20 fold using acetonitrile:water (75:25; v/v) solution containing 5 mM Tris (pH 8.0) and 0.25% (v/v) triethylamine (DEA). To a 100 µL of above diluted homogenate, 80 µL of internal standard (80 ng/mL of stable labeled ^15^N_5_-AMP, ^15^N_5_-ADP, and ^15^N_5_-ATP). The samples were further precipitated with 320 µL of acetonitrile:water (75:25; v/v) solution containing 5 mM Tris (pH 8.0) and 0.25% (v/v) DEA. After vortex-mixing and centrifuging, five µL of supernatant were then injected into the LC-MS/MS system for analysis.

LC-MS/MS Procedure: Separations were carried out on a Luna Amino (NH_2_) column (Phenomenex, Torrance, CA) 50 mm × 2.0 mm, packed with 3.0 μm particles. Two Shimadzu LC-20 AD binary pump system (Columbia, MD) and a Leap Technologies HTS PAL autosampler (Carrboro, NC), Valco column switching valve (VICI, Huston, TX) and an API 4000 tandem quadrupole mass spectrometer (Concord, Ontario, Canada) with a TurboIonspray™ source operating in negative-ion multiple reaction monitoring mode were used. A binary gradient at a flow rate of 0.35 mL/min was used to perform the separations. Mobile phase A consisted of 100 mM hexafluoro-2-isopropanol (HFIP) and 0.5% DEA (v/v) in water, and mobile phase B consisted of 100 mM HFIP and 0.5% DEA (v/v) in acetonitrile. A 5.0 μL injection of each sample was loaded onto the column, separated, and eluted using the following gradient conditions (time (min), % mobile phase B): (0, 75) (0.8, 75) (3.0, 58) (3.50, 60) (3.51, 30) (5.0, 30) (5.01, 75) (6.0, 75). The column eluent was directed into a Sciex API 4000 mass spectrometer. The ESI (negative ionization mode) conditions were optimized as follows: entrance potential (EP) −10 V, curtain gas (CUR) 25, collision gas (CAD) 12, ionspray voltage (IS) −4000 V, nebulizer gas (GS1) 60, auxiliary gas (GS2) 60, temperature (TEM) 600 °C. The instrument was operated using multiple reaction monitoring (MRM) based on the formation of the most abundant product ion for AMP (346 → 79; DP: -60 V; CE: -50 V; CXP: -6 V), ADP (426 → 79; DP: -60 V; CE: -60 V; CXP: -6 V) and ATP (506 → 159; DP: -80 V; CE: -40 V; CXP: -10 V).

*NMR Analysis.* Frozen liver tissue samples (50-100 mg) were placed in 2 mL centrifuge tubes containing 600 μL of cold 2:1 methanol/water solution and lysed using Tissue Lyser II (Qiagen, Valencia, CA) for 90 second at 20 Hz. Then the mixture was sonicated for 60 second in ice cold water. The homogenized mix was then centrifuged at a speed of 11,180 g at 4 °C for 10 min and the supernatant was collected. The homogenization was repeated two more times and the supernatant ( ~1.8 mL in total) was mixed together and then lyophilized in a CentriVap concentrator (Labconco, Kansas City, MO) for NMR spectroscopic analysis.

Liver tissue aqueous extracts were reconstituted to 160 mg/mL in deuterated water containing 100 mM phosphate or tris buffer (pH = 7.4) for ^1^H and ^31^P NMR experiments respectively. 0.5 mM sodium (3-trimethylsilyl)-2,2,3,3-tetradeuterio-propionate (TSP) or triethyl phosphate was added for ^1^H or ^31^P chemical shift and concentration reference. ^1^H NMR spectra were recorded on a Bruker Avance III 600 MHz spectrometer with TCI cryoprobe at 25 °C using standard one dimensional nuclear Overhauser effect spectroscopy (NOESY) pulse sequence with presaturation of solvent signal. A mixing time of 100 ms was applied, during which water signal can be suppressed further. 32K data points were acquired with a 7000 Hz sweep width, 5.3 s repetition time and 512 scans. Spectra were processed and analyzed by using Chenomx NMR Suite 7.6. The concentrations of 55 metabolites were identified and quantified by targeted profiling using the library provided by Chenomx. ^31^P NMR spectra were recorded at 15 °C on a Bruker Avance III HD 500 MHz spectrometer equipped with prodigy probe. 16K data points were acquired with a 8000 Hz sweep width, 2 s repetition time and 8K scans. The data set was zero-filled to 32 K and an exponential line-broadening of 1 Hz was applied to the free-induction decay prior to Fourier transformation. Spectra were processed and analyzed by MestReNova.

1. Zhang G, Walker AD, Lin Z, Han X, Blatnik M, et al. (2014) Strategies for quantitation of endogenous adenine nucleotides in human plasma using novel ion-pair hydrophilic interaction chromatography coupled with tandem mass spectrometry. J Chromatogr A 1325: 129-136.
